# Physical disruption of intervertebral disc promotes cell clustering and a degenerative phenotype

**DOI:** 10.1038/s41420-019-0233-z

**Published:** 2019-12-17

**Authors:** Polly Lama, Harry Claireaux, Luke Flower, Ian J. Harding, Trish Dolan, Christine L. Le Maitre, Michael A. Adams

**Affiliations:** 1 Department of Anatomy, Sikkim Manipal Institute of Medical Sciences, Sikkim Manipal University, Sikkim, India; 2 Nuffield Department of Orthopaedics, Rheumatology and Musculoskeletal Sciences, University of Oxford, Oxford, UK; 3 Imperial College Healthcare, NHS Trust, London, UK; 4 Department of Orthopaedics, Southmead Hospital, University of Bristol, Bristol, UK; 5 Centre for Applied Anatomy, University of Bristol, Bristol, UK; 6 Department of Cell Biology & Tissue Regeneration, Sheffield Hallam University, Sheffield, UK

**Keywords:** Molecular modelling, Diseases

## Abstract

To test the hypothesis that physical disruption of an intervertebral disc disturbs cell-matrix binding, leading to cell clustering and increased expression of matrix degrading enzymes that contribute towards degenerative disc cell phenotype. Lumbar disc tissue was removed at surgery from 21 patients with disc herniation, 11 with disc degeneration, and 8 with adolescent scoliosis. 5 μm sections were examined with histology, and 30-µm sections by confocal microscopy. Antibodies were used against integrin α5beta1, matrix metalloproteinases (MMP) 1, MMP-3, caspase 3, and denatured collagen types I and II. Spatial associations were sought between cell clustering and various degenerative features. An additional, 11 non-herniated human discs were used to examine causality: half of each specimen was cultured in a manner that allowed free ‘unconstrained’ swelling (similar to a herniated disc in vivo), while the other half was cultured within a perspex ring that allowed ‘constrained’ swelling. Changes were monitored over 36 h using live-cell imaging. 1,9-Di-methyl methylene blue (DMMB) assay for glycosaminoglycan loss was carried out from tissue medium. Partially constrained specimens showed little swelling or cell movement in vitro. In contrast, unconstrained swelling significantly increased matrix distortion, glycosaminoglycan loss, exposure of integrin binding sites, expression of MMPs 1 and 3, and collagen denaturation. In the association studies, herniated disc specimens showed changes that resembled unconstrained swelling in vitro. In addition, they exhibited increased cell clustering, apoptosis, MMP expression, and collagen denaturation compared to ‘control’ discs. Results support our hypothesis. Further confirmation will require longitudinal animal experiments.

## Introduction

Intervertebral discs are pads of fibrocartilage lying between vertebral bodies in the spine. They allow some intervertebral movement and distribute compressive loading evenly on the adjacent vertebral bodies. Discs comprise a soft centrally located nucleus pulposus surrounded by a tough annulus fibrosus, with a thin hyaline cartilage ‘endplate’ lying above the disc and each adjacent vertebral body. Adult discs are normally avascular and aneural, and cellularity is very low except in the peripheral annulus^1,2^.

Disc ‘degeneration’ is common in the human spine. It has been defined as a cell-mediated response to structural failure, as the small cell population attempts vainly to repair an extensive cross-linked^2,3^. This concept has widespread support^4,5^ and explains animal ‘injury’ models of disc degeneration^5–7^. Macroscopically, a degenerated disc contains annulus fissures^8^, and/or endplate defects^9^, and microscopic changes include accelerated loss of water-retaining glycosaminoglycan (GAG) molecules^10^, nerve and blood vessel infiltration^11^, cell clustering^12^, and upregulation of matrix-degrading enzymes^13^. Major risk factors include genetic inheritance^14^, age, and excessive physical activity^15,16^. Structural features of disc degeneration are strongly associated with chronic back pain, including radial fissures in the annulus^17^ and defects in the endplates^18^, although typical age-related changes (such as GAG loss and minor bulging) are not^10,19^.

A disc ‘herniation’ represents a particular type of degeneration in which part of the nucleus is displaced into, or through, a radial fissure in the annulus, often taking some annulus or endplate with it. In life, this can result from excessive or repetitive mechanical loading^20,21^, and discs are intrinsically most vulnerable to herniation in middle-age, following moderate (but not severe) degenerative changes^6,22^. Herniated tissue can impinge on spinal nerves and cause distressing symptoms (‘sciatica’) radiating to the buttock or leg. Disc herniation can initiate further degenerative changes, because displaced nucleus and annulus tissue swells by 100–300% within a few hours, losing much of its GAGs^23,24^. Blood vessels and nerves grow into this GAG-depleted and free-swelling tissue especially inside annulus fissures^8,11,14^. Inflammatory cells^25^ and bacteria^26^ can similarly invade a herniated disc and contribute to discogenic pain. Because these adverse changes arise from initial swelling of displaced tissue, they do not occur to such an extent in discs that degenerate in situ without herniating^11,23^.

Other characteristic changes in disc herniation, particularly cell clustering and upregulation of matrix-degrading enzymes, may also be consequences of initial tissue disruption and swelling. They follow disc injury in animal models^22^, although the small and young animals used in such experiments are not always a reliable guide to disc degeneration in humans^22,23^, for whom no equivalent data are available. Therefore, we sought evidence that in mature human intervertebral discs, matrix disruption and swelling can disturb cell-matrix binding and lead to cell clustering, together with expression of a degenerative cell phenotype.

Two complimentary studies were performed. The first, on surgically retrieved human discs, aimed to show close and consistent spatial associations between matrix fissures, focal GAG loss, decreased cell-matrix binding, cell clustering, and expression of matrix-degrading enzymes. The second study involved tissue culture and aimed to provide experimental evidence of a causal chain between some of these features.

## Results

### Live-cell imaging in explants

During the first 6 h, unconstrained disc tissues swelled rapidly, increasing the size of unconstrained disc explant (viewed area) by ~100–150%. The swelling capacity of the unconstrained and constrained IVD tissue was assessed after 36 h by using the DMMB analysis which measured the amount of GAG released during the process of tissue swelling in the two conditions IVD tissue were placed. Rapid swelling and release of GAGs prevented clear visualisation of cell nuclei. Tissues constrained by the perspex ring showed minimal swelling. After 12 h, unconstrained tissue swelling slowed and allowed clearer visualisation of cells and matrix. Time-lapse recording showed rotational cell movements occurring at irregular time points. Clusters of blue-stained cell nuclei were more common in unconstrained than constrained tissue, but this observation could not be quantified reliably within the cubed tissue explants. At 36 h, unconstrained tissue shrunk after losing its GAG content showing a loss of volume which complimented DMMB analysis, cell/tissue movements declined during this process and a typical time-lapse recording from an unconstrained sample are shown in the Supplementary Data.

### DMMB assessments of GAG loss from explants

Curve-fitting of data from standard solutions yielded a linear calibration, with *r*^2^ = 0.913 (*P* < 0.001). This was used to show (Table 1) that unconstrained explants were free to swell and release almost twice as much GAGs into the medium than constrained explants (*P* < 0.01).

**Table 1:** Summary of numerical results.

Variable (scale/units)	Explant—constrained (2)	Explant—unconstrained (3)	Variable (scale/units)	Non-degenerated (5)	Herniated (6)
Tears (0–3)	1.3 (0.3)*	1.9 (0.2)*	Tears (0–3)	1.1 (0.3)**	2.2 (1.5)**
GAG loss (0–3)	1.4 (0.4)*	2.0 (0.2)*	GAG loss (0–3)	1.3 (0.5)*	2.1 (0.4)*
Cell clustering (0–5)	1.2 (0.3)	1.5 (0.6)	Cell clustering (0–5)	1.2 (0.9)*	2.5 (1.0)*
GAG loss (DMMB μl/ml)	0.048 (0.031)**	0.090 (0.030)**	Blood Vessels (0–3)	0 (0)**	1.3 (1.1)**
MMP-1 (cells/mm^2^)	14.8 (12.0)*	34.5 (29.2)*	MMP-1 (cell/mm^2^)	13.5 (9.7)**	99.8 (71.5)**
Integrin α5β1 (cells/mm^2^)	17.5 (15.6)*	33.4 (20.4)*	MMP-3 (cell/mm^2^)	11.1 (11.0)**	84.4 (79.1)**
Denatured collagen I, II (area µm^2^)	820 (1201)**	7716 (5426)**	Caspase-3 (cell/mm^2^)	10.0 (6.3)**	67.2 (45.2)**

Values refer to the mean (STD). Significant differences are shown in bold, with significance levels denoted

^a^Columns 2 and 3 compare constrained and unconstrained explants

^b^Columns 5 and 6 compare non-degenerated (‘control’) and herniated discs

*P < 0.05

**P < 0.01

### Other comparisons of constrained and unconstrained explants

**Figure 1:**
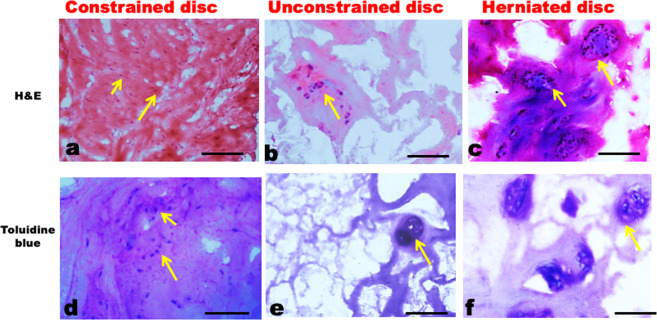
Histological comparisons of np/iaf regions after 36 h with haematoxylin & eosin (H&E) and toluidine blue dye. Constrained (**a**–**d**) and unconstrained disc (**b**–**e**) and its comparisons with herniated discs (**c**–**f**). Arrows show cells and clusters, especially around disrupted regions. Scale bar = 100 µm.

**Figure 2:**
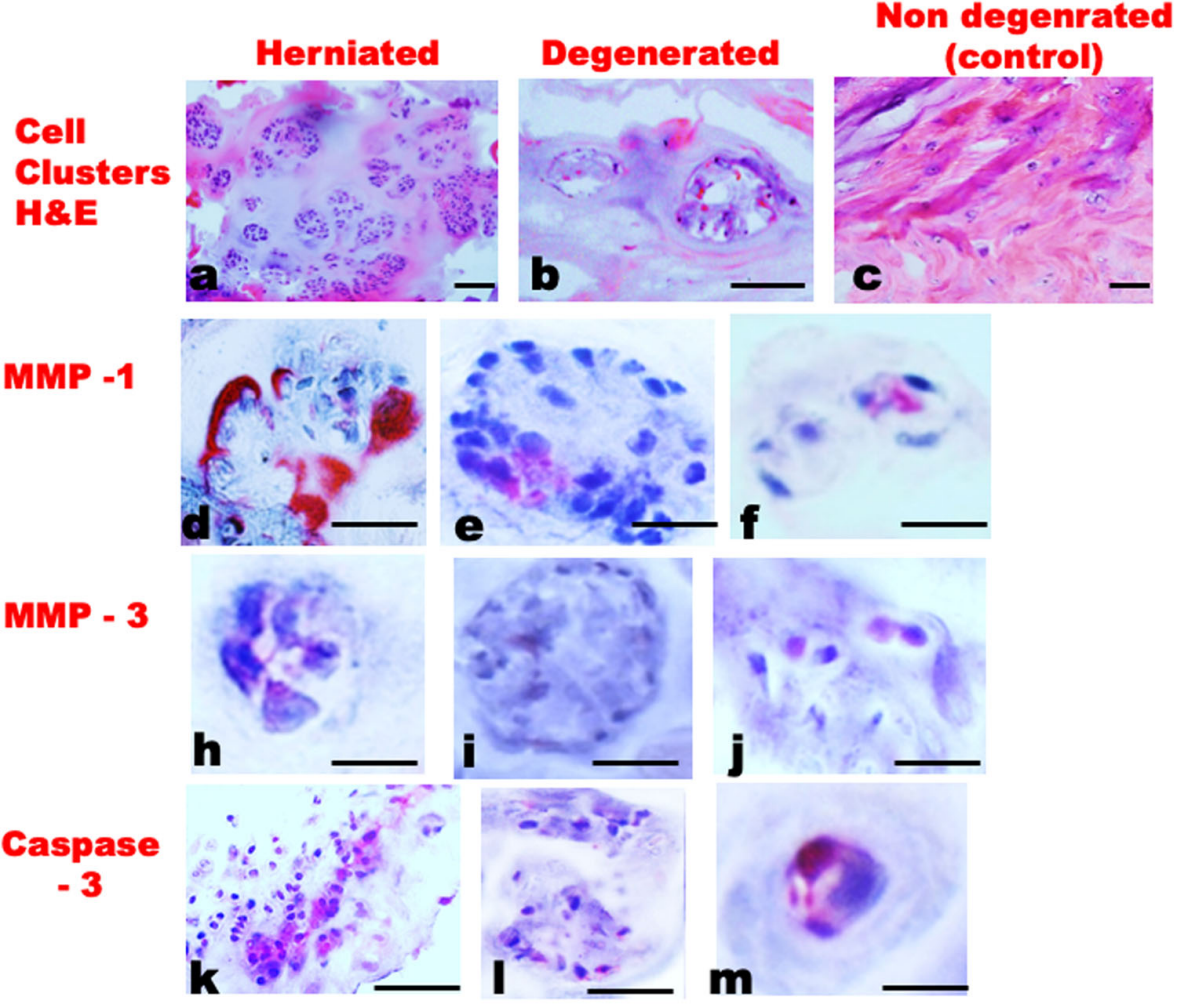
Immunohistochemistry images comparing herniated, degenerated, and non-degenerated (control) discs. Five-micrometre-thin sections stained with H&E to show cell clusters in three conditions (**a**–**c**). Red-staining antibodies refer to: MMP-1 (**d**–**f**); MMP-3 (**h**–**j**); Caspase-3 (**k**–**m**). Scale bar = 50 µm.

**Figure 3:**
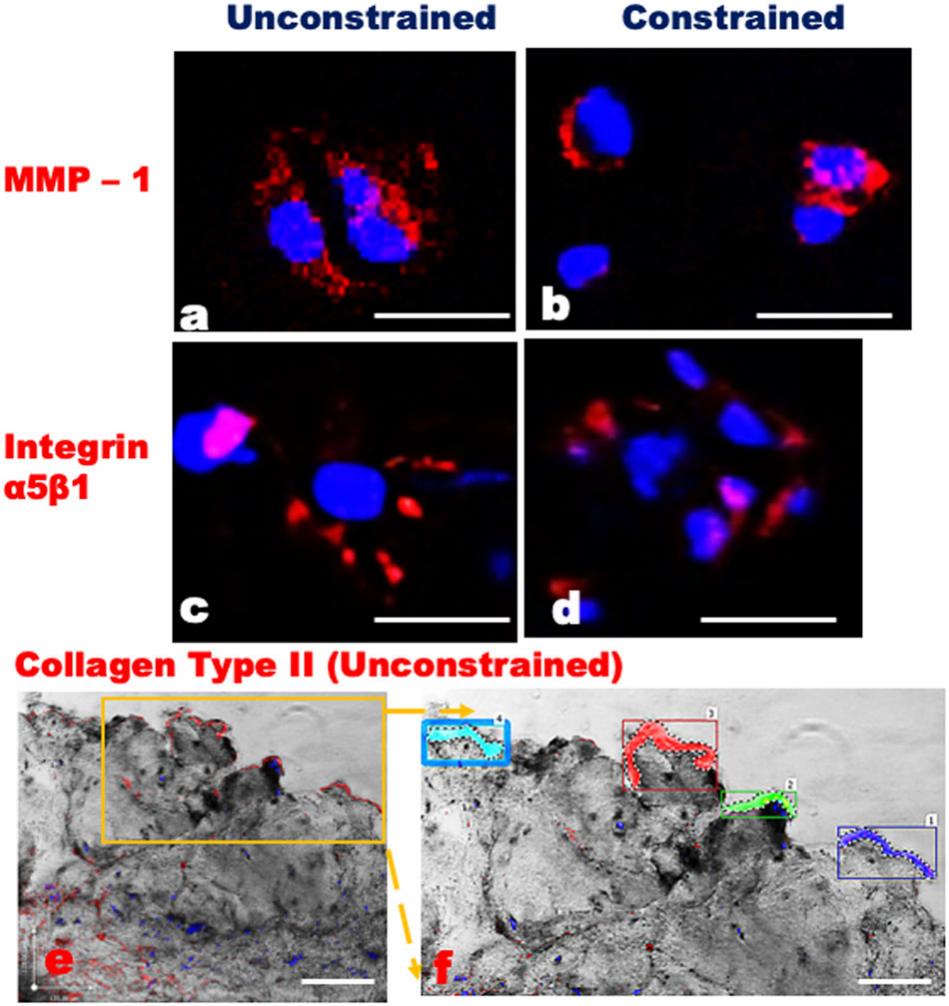
Immunofluorescence staining of thick (30 µm) sections viewed on a confocal microscope, comparing constrained (right) and unconstrained (left) disc explants. Red-staining for antibodies refer to: MMP-1 (**a**, **b**), integrin α5β1 (**c**, **d**), and denatured collagen type II (**e**). Cell nuclei are stained blue with Dapi. In **f**, ‘Volocity’ image analysis software was used to measure the areas of denatured collagen, which colour coded and analysed positive stained area in 30-µm-thick section as clear blue, red, green, and blue colour. Scale bar = 50 µm.

Histological evaluations after 36 h (Fig. 1) indicated that unconstrained tissues contained more fissures and tears, lost more GAG (especially near fissures and surfaces), and contained slightly more cell clusters, although this last result did not reach significance (Table 1). Unconstrained tissues exhibited a loss of matrix integrity that persisted as wide displacements between collagen lamellae, with clear loss of toluidine blue staining (Fig. 1b–e), which has a high affinity for staining GAGs^21,27^. In contrast, constrained samples, at the inner annulus/nucleus junction showed closely packed collagen lamellae, well-aligned fibroblasts, and rounded inner annular cells (Fig. 1a–d). Confocal and immunostaining analysis showed increased expression of matrix metalloproteinase 1 (MMP-1) in unconstrained explants compared to constrained (Table 1). MMP-1 was located within the cytoplasm, cell membranes, and pericellular matrix, suggesting its existence in both activated and latent forms (Fig. 2d–f). In both constrained and unconstrained tissues, cells and cell clusters positive for MMP-1 were mostly located within disrupted and GAG depleted regions (Figs. 2d–f, 3a–d). Exposed integrin α5β1 receptors in 30-µm-thick sections were twice as frequent in unconstrained tissue as in constrained (Table 1). Groups of these exposed receptors often surrounded a cell (Fig. 3c)^28,29^ in swollen unconstrained tissue but they were less evident in constrained tissue (Fig. 3d). Denatured collagen (Types I and II) was mostly located around the periphery of unconstrained disc tissue that was free to swell and disrupt itself (Fig. 3e), and the area occupied by denatured collagen (Fig. 3f) was increased ninefold in unconstrained compared to constrained explants (Table 1).

### Comparison of herniated, degenerated, and scoliotic (‘control’) discs

**Figure 4:**
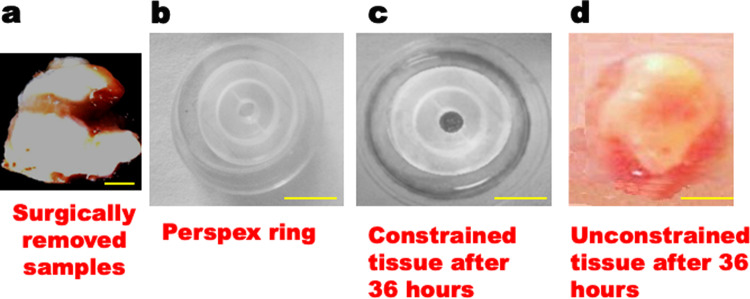
Visual comparisons between constrained and unconstrained disc tissue. **a** Surgically removed intervertebral disc tissue. **b** Perspex ring used for constraining the 5 mm^3^ disc tissue blocks. **c** Disc tissue in the perspex ring as ‘constrained tissue’ that was refrained from swelling for 36 h. **d** ‘Unconstrained’ free swelling disc after 36 h.

**Figure 5:**
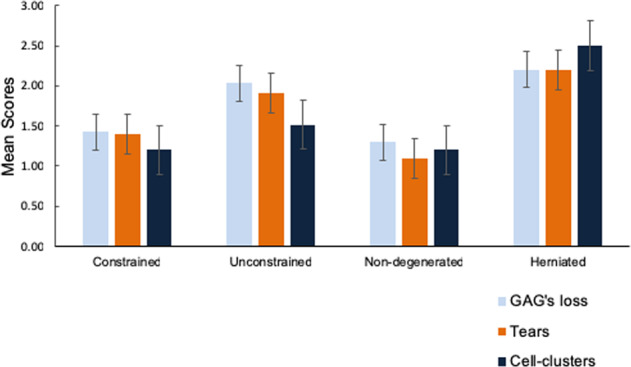
Histological variables are compared between ‘constrained’ and ‘unconstrained’ disc explants, and also between ‘non-degenerated’ (control) and ‘herniated’ tissues. Variables are GAG loss (scored 0–3), matrix tears (0–3), and cell clustering (0–5). Error bars indicate the SEM. Comparisons suggest that the processes of disc swelling in vitro and herniation in vivo have similar effects on tissue histology. Summary of significance between the variables assessed is shown in Supplementary Table 1.

Differences between non-degenerated (control) and herniated tissues were similar to differences (described above) between constrained and unconstrained explants (Figs. 4, 5). This was confirmed by semi-quantitative histology scores, which clearly showed herniated discs with increased matrix tears, GAG loss, cell clusters, and blood vessels (Table 1, columns 5 and 6). Large cell clusters contained as many as 60 cells within herniated discs (typically 5–30 cells). They were located near the nucleus/inner annulus junction, especially in regions with disrupted lamellae or fissures and loss of GAG staining (Figs. 1c–f, 2a–c). Large cell clusters were not common in scoliosis (‘control’) discs. MMP-1 and MMP-3 immuno-positive cells and cell clusters were abundant in fissured and disrupted regions in herniated and degenerated discs (Fig. 2d–j) in comparison to (scoliosis) controls, and thus were comparable to unconstrained explant tissues (Fig. 5). Caspase-3 activity was seen in cell clusters and in single cells (Fig. 2k–m) and was much more common in herniated discs compared to non-degenerated controls (Table 1) and was similar to observations made in constrained and unconstrained explants (numerical data excluded).

## Discussion

### Summary of results

Human intervertebral disc explants swelled rapidly in saline, distorting the matrix and creating relative movement between disc cells. After 36 h, free-swelling explants showed greater loss of GAGs, increased exposure of integrin binding sites, greater production of MMP-1 and MMP-3, and increased collagen denaturation, compared to explants whose swelling was partially constrained. Comparisons between herniated and non-degenerated (scoliotic) disc tissues showed differences that resembled those between ‘free swelling’ and ‘partially constrained’ explants (Figs. 2, 3), suggesting close parallels between the processes of disc herniation and unconstrained swelling. In addition, herniated discs showed more cell clustering and apoptosis than even degenerated discs. Overall, results indicate that physical disruption of the disc matrix can lead to focal swelling, GAG loss, disturbed cell-matrix binding, cell clustering, and a degenerative cell phenotype.

### Justification for primary antibodies used

MMPs 1 and 3 play an essential role in local proteolysis of the extracellular matrix, including collagens^11^. MMPs 1 and 3 in particular are associated with disc collagen damage^21,30^. Collagen types I & II are abundant in the disc, and their denaturation is indicative of proteolytic cleavage^7^ and mechanical overloading^2,6^. Uncoiled, fragmented, and denatured collagens can also initiate inflammation in wounded tissue^25^. Therefore, increased collagen denaturation following free swelling can be interpreted as indicative of matrix disruption and a degenerative phenotype^7,31^. Integrin α5β1, a receptor for fibronectin, mediates interactions between the cell surface (through actin filaments) and the immediate surrounding matrix, and is involved in the initiation of mechanotransduction in intervertebral disc cells^32^. Identifying exposed α5β1 receptors in free swelling tissue is therefore indicative of disrupted cell–matrix interactions and may indicate a degenerative cell phenotype. Caspase-3 is involved in the activation cascade in apoptosis, so increased caspase-3 activity can act as a marker for DNA fragmentation and apoptosis in injured and degenerated disc tissue^33^.

### Strengths and weaknesses of the study

Comparisons between herniated, degenerated, and non-degenerated (scoliotic) discs benefited from the use of human tissues that were believed to be painful, and they were examined using techniques (including immunofluorescence and confocal microscopy) that yielded high-resolution images of degenerative features, including exposed integrin binding sites (Fig. 3), deep within the tissues. Because this comparison was cross-sectional, it could only infer causation from spatial associations. However, the controlled experiment on disc explants, although limited in scope, showed that unconstrained disc swelling can disturb integrin binding and lead to increased expression of matrix-degrading enzymes, providing support for two key steps in our hypothesis. The perspex ring reduced but did not eliminate disc swelling, but this is not a problem because vertical disc swelling in life is only modified (but not eliminated) by applied mechanical loading^34^. Elimination of disc swelling would have required the perspex ring to restrain the disc specimens on all sides, and this would have interfered with metabolite transport to cells within the explant^26^. The DMMB assay^35^ provided precise measures of GAG release which complemented the semi-quantitative grading of GAG loss used in the cross-sectional comparison of herniated and degenerated discs. Similarly, the Volocity image analysis software allowed precise quantification of several histological variables, including collagen denaturation.

### Relationship to previous work

Cell clusters are common in degenerated discs, especially those that are herniated^36^, and are associated with cell proliferation^12,37^ and with progenitor cells^38^. Cluster formation can be influenced in vitro by cell density and availability of nutrients^39^. Cell clusters may represent a repair response because they are usually found within disrupted nucleus or inner annulus tissue^40^ where in-growing nerves and blood vessels also are present^8,11^. Clustering cells increase the expression of matrix-degrading enzymes^11,21,41^ and a relatively high proportion of them are senescent^42,43^. A previous comparison of degenerated and herniated disc tissues showed the latter to have greater GAG loss, neovascularisation, innervation, cellularity, and expression of MMPs than discs that degenerated without herniating^21^.

### Explanation of results

The living human spine is habitually loaded by gravity and/or by tension in muscles and ligaments^44,31^, so the natural tendency for intervertebral disc GAGs to swell up in tissue fluid^30^ is largely restrained. This balance between tissue swelling and mechanical restraint explains why diurnal variations between night-time rest and daytime activity cause a 20% variation in disc hydration^45^. Disc herniation, however, permanently unloads any displaced tissue fragments, allowing them to swell by 200–300% in just a few hours^41,43^. Gross swelling is typically followed by rapid shrinking during the following days as GAGs (or their fragments) diffuse out of the disrupted and swollen tissue. At a cellular level, these changes may slow down anabolic processes such as GAG synthesis^34,36–40^ and upregulate catabolic processes including the synthesis of matrix-degrading enzymes such as the MMPs^46,47^. A combination of focal matrix distortion and activated MMPs could cause some disc cells to lose their weak integrin binding: for example, in the present study exposing receptors for α5β1 integrin (Fig. 3c) which bind disc cells to fibronectin in the matrix^47^. Impaired binding could explain altered integrin mechanotransduction in degenerated discs^28^, and also the presence of fibronectin fragments in the matrix which have been shown to induce catabolic changes in disc cells^48^. Cell–cell binding can promote clustering in some disc cells^49,50^ so it is conceivable that disturbed cell-matrix binding could have a similar effect. Cell clusters have the potential to regenerate and repair a damaged disc, although the heavily cross-linked matrix in adult human discs^51^ may frustrate even the largest clusters and lead instead to increased cell senescence^39,40^ and apoptosis as represented here by the cell death marker Caspase-3^52,29^. Increased antibody binding to denatured collagen in unconstrained (or herniated) tissue (Fig. 3e, f) may be attributable to MMPs (and possibly abnormal swelling) degrading the triple helical regions of fibrillar collagens^53^, and to increased exposure of collagens to MMPs following GAG loss.

### Clinical relevance

This study suggests how focal damage to an intervertebral disc might lead to loss of proteoglycans, collagen disruption and denaturation, and disturbed cell-matrix binding. These changes in turn could lead to cell clustering, and a degenerative disc cell phenotype (Fig. 5). Disc damage may be hard to avoid, especially in some occupations, but it should be possible to modulate cellular responses to this damage, and in particular, to block the formation of large cell clusters or the matrix-degrading enzymes synthesised by them.

## Conclusions

Disrupted and herniated disc tissue swells and loses GAGs. The resulting distortion of the matrix can disturb cell-matrix binding, leading to cell cluster formation and a degenerative disc cell phenotype.

## Materials and methods

### Specimen collection

After ethical clearance (NReS, Frenchay Hospital, Bristol, UK), disc samples were removed at surgery from 11 patients undergoing spinal fusion for back pain believed to originate from non-herniated discs. Additional disc specimens were obtained from 40 patients undergoing surgery at Southmead, Frenchay or Spire hospitals in Bristol: 21 of these patients had a disc herniation, 11 had severely degenerated but non-herniated discs, and 8 had adolescent idiopathic scoliosis (AIS) with spinal curvature <48°. These latter discs served as young ‘non-degenerated’ controls. An anonymous clinical data sheet was obtained for each patient, as well as an MRI scan which allowed Pfirrmann^24^ grade of disc degeneration to be assessed. Clinical data are summarised in Supplementary Table a.

### Tissue culture

Each of the 11 non-herniated samples was divided into two small tissue blocks (~5 mm^3^), which were cultured (37 °C, 5% CO_2_) in a Mat-Tek petridish containing Dulbecco’s Modified Eagle Medium (DMEM) with 5 ml/l penicillin (Thermo Fisher Scientific, UK) and 2.5 ml/l Gibco amphotericin B (Thermo Fisher Scientific, UK). One block of each pair was allowed to swell without restraint; the other was restrained in a sterile perspex ring (Fig. 4a–d) that allowed vertical but not radial swelling, which is similar to the restraint imposed by a healthy annulus. The perspex rings were provided by Professor Christine Le Maitre, Sheffield Hallam University, UK^52^.

### Live cell imaging

Nuc-Blue live cell nuclear stain (Invitrogen, UK) was applied to the constrained and unconstrained tissue blocks in order to visualise cell nuclei. A phase contrast background was used to locate cell nuclei within the surrounding matrix. Tissue blocks were observed under 20× objective in a wide-field microscope with incubator for maintaining the standard culture conditions. ‘Volocity’ 3D image analysis software was used to record time-lapse video of all disc samples at 6, 12, and 36 h.

### DMMB assay for tissue GAG loss

The total GAG content of the tissue medium following constrained or unconstrained swelling was quantified using the 1,9-di-methyl methylene blue (DMMB) colorimetric assay^35^. The assay served as a surrogate measure for assessing tissue swelling, as intervertebral disc has high content of GAG, it is known that structural changes result in degeneration of disc which is preceded and accompanied by loss of GAG’s. In unconstrained explants, GAGs readily leach out into the tissue medium when they lose their structural integrity after rapid swelling. Thus, to assess the loss of GAGs, bovine chondroitin sulphate standards from 0 to 300 µl/ml concentrations were used for calibrations. 10 μl of the chondroitin sulphate was added to 100 µl of the DMMB dye with suitable blanks. A total of 2.5 ml of the constrained and unconstrained tissue medium collected after 36 h of the experiment was digested with papain and diluted in phosphate buffer with ethylene-di-amine tetra acetic acid (EDTA). Formic acid was used to adjust the pH to 3. Ten microlitres of the digested tissue medium was then mixed with 100 µl of the DMMB dye. Five minutes after adding the DMMB dye, absorbance was measured at A_525_ nm with a spectrophotometer.

### Histology

All tissues samples, including constrained and unconstrained explants after swelling, were snap frozen by immersing in chilled iso-pentane that was cooled with liquid nitrogen. This step minimised the risk of tissue damage from freeze fracture. Samples were then stored at −80 °C. When required, the samples were embedded in optimal cutting tissue medium before sectioning in a Leica CM1900 cryostat (Heidelberger, Nussloch, Germany) at a thickness of 5 µm. Thin sections were post-fixed in 10% neutral buffered formalin. Haematoxylin & eosin (H&E) stain was used for scoring degenerative changes, while toluidine blue stain was used for assessing GAG (proteoglycan) loss. Three fields of views from each sample were graded according to the modified Boos method, using ordinal scales of 0–3 or 0–5^8,54^.

### Immunohistochemistry and immunofluorescence

Parts of each snap-frozen tissue block (including constrained and unconstrained explants) were sectioned at 5 µm and 30 µm thickness in the same cryostat, and post-fixed in acetone for 10 min at −20 °C. Both thick and thin sections were used to facilitate the identification and follow up on fine linear matrix structures, and cell clusters, respectively.

Thick sections were incubated overnight at 4 °C, washed in PBS, and non-specific binding sites were blocked by application of donkey serum (Sigma Aldrich, UK) at 4 °C for 1 h, at 1:5 dilutions, in PBS. Sections were washed again in PBS and the following primary antibodies were applied: MMP-1 (Abcam, UK), integrin α5β1 (Abcam, UK), denatured collagen types I and II (EndMillipore, USA), and caspase-3 (Invitrogen, UK). All antibodies were used at 1:50 dilutions in PBS, and PBS alone was used for controls. Choice of primary antibodies is justified in the Discussion. Donkey anti-mouse alexa 594 secondary antibody (Invitrogen, UK) was applied for 1 h at 1:250 dilution, and auto-fluorescence was quenched using 0.01% Sudan Black B. Nuclei were counter-stained with Vecta Shield Dapi (Vector, UK).

Thin sections were fixed in neutral buffered formalin, blocked in rabbit serum (Dako, UK) and washed in PBS. Sections were then applied with primary antibodies to MMP-1 (Abcam, UK) and MMP-3 (Millipore, UK) at 1:50 dilutions in PBS, and with caspase-3 (Invitrogen, UK) at 1:100 dilutions in PBS. Antibodies were omitted from controls. Thin sections were incubated overnight, washed in PBS, and incubated with biotinylated rabbit anti-mouse secondary antibody (Dako, UK) at 1:200 dilutions for 1 h. Following washes in three changes of PBS, antigen–antibody signal was amplified with extra avidin alkaline phosphatase conjugate (Sigma Aldrich, UK) applied at 1:100 dilution for 1 h. After three more rinses in PBS, fast red chromogen (Sigma Aldrich, UK) was applied, diluted in distilled water. Nuclei were counterstained with Mayer’s Haematoxylin, and sections were mounted with aqueous faramount mounting medium (Dako, UK).

### Quantitative analysis of confocal microscope images

30 μm sections were sequentially scanned with a Leica SP5-AOBS confocal laser scanning microscope attached to a Leica DM 16000 inverted epifluorescence microscope. To prevent crosstalk between different secondary fluorophores, and photo bleaching, a 4-line average was used for each image. Four thick sections were analysed for each tissue block (11 constrained, 11 unconstrained). Volocity 3D image analysis software (Perkin Elmer, UK) was used to count the number of positively stained cells, to identify exposed integrin receptors, and to calculate the total cross-sectional areas occupied by denatured collagen types I and II.

Eight thin (5 µm) sections were analysed from each block. Positively stained cells were counted using a Leica DM6000B fluorescence microscope (Leica, UK) attached with an Olympus DP72 12.8 megapixels camera with a fixed frame view of 650 µm × 500 µm. The number of cells and cell clusters, which stained for MMP1, MMP3, and caspase-3, were counted across the entire section at 20× magnification, following a frame by frame shift from left to right. An average of eight fixed frames were viewed per thin section. Total numbers of immunoreactive cells were counted manually and re-analysed using the volocity image analysis software.

### Statistical analysis

Scores from individual fields of view and sections were averaged to yield quantitative data. Differences between herniated, degenerated, and scoliosis tissues were assessed by ANOVA. Constrained and unconstrained tissues were compared using matched-pair *t*-tests. All the statistical tests were performed using SPSS software v.18, and a *P* < 0.05 was considered statistically significant.

## Supplementary information

Supplementary table a

Video a

Video b

Supplemental Material File #1
