# Supplementary material for: Physical disruption of intervertebral disc promotes cell clustering and a degenerative phenotype
Source: Cell Death Discov. 2019 Dec 17;5:154. doi: 10.1038/s41420-019-0233-z (PMC6917743; doi:10.1038/s41420-019-0233-z)
Supplement: Supplementary file 1 — Supplementary table a [file 41420_2019_233_MOESM1_ESM.docx]

**Supplementary table 1**: Details of surgically removed intervertebral disc tissues.

|  | **Herniated discs** | **Degenerated discs** | **Non-degenerated (scoliotic) discs** | **Degenerated discs (explants)** |
| --- | --- | --- | --- | --- |
| n | 21 | 11 | 8 | 11 |
| Age (yrs) | 53 (35 - 74) | 53 (39 - 72) | 14.5 (14-15) | 44 (33-55) |
| Spinal level (n) | L2-3 (1), L3-4 (2), L4-5 (4), L5-S1 (14) | L2-3 (1), L4-5 (6), L5-S1 (4) | T12-L1 (1), L1-2(2), L2-3(2), L3-4(2), L4-5(1) | L3-4 (2), L4-5 (6), L5-S1 (3) |
| Gender | M (8), F (13) | M (6), F (5) | F (8) | M (4), F (7) |
| Pfirrmann grade | 3.6 (3 - 4) | 3.0 (2 - 4) | 1 | 3.8 (3-5) |
